# Clinical characteristics and decreased CD4^+^CD25^+^Foxp3^+^ regulatory T cells and IL-35 in pediatric immune thrombocytopenia in a single center

**DOI:** 10.3389/fimmu.2026.1782560

**Published:** 2026-03-10

**Authors:** Yan Yang, Dan Lu, Bei Wu, Lijun Wan, Xiaoyun Zhan, Alan Zhao, Pei Tan, Buyun Shi, Juan Huang, Yinghong Lu

**Affiliations:** 1 Department of Pediatric Hematology, Maternal and Child Health Hospital of Hubei Province, Tongji Medical College, Huazhong University of Science and Technology, Wuhan, China; 2 Department of Laboratory medicine, Maternal and Child Health Hospital of Hubei Province, Tongji Medical College, Huazhong University of Science and Technology, Wuhan, China; 3 Department of Pediatric Intensive Care Unit, Maternal and Child Health Hospital of Hubei Province, Tongji Medical College, Huazhong University of Science and Technology, Wuhan, China

**Keywords:** cytokines, descriptive epidemiology, IL-35, pediatric immune thrombocytopenia, regulatory T cells

## Abstract

**Background:**

Immune thrombocytopenia (ITP) exhibits distinct epidemiological characteristics in terms of age and sex distribution. In contrast to adult ITP, the clinical features and immunological mechanisms underlying pediatric ITP remain incompletely understood.

**Objective:**

To analyze the clinical characteristics, treatment response and immunological profiles of newly diagnosed pediatric ITP patients, in order to provide a reference for clinical diagnosis and treatment.

**Methods:**

This study enrolled 240 newly diagnosed pediatric ITP patients. Demographic characteristics, predisposing factors, clinical manifestations, treatment response, and prognosis were analyzed. Lymphocyte subsets were assessed by flow cytometry, and serum cytokine levels were measured using ELISA.

**Results:**

The <1 year age group constituted the largest subgroup (51.67%). Gender distribution differed significantly across the age groups (*p* = 0.005), with a marked male predominance in <1 year group (male-to-female ratio: 2.35:1). A history of vaccination within 1–4 weeks prior to onset was reported in 12.08% of patients, predominantly in the <1 year group (19.35%), whereas 24.58% had a recent history of respiratory infection, most frequently in the 3–6 years group (61.11%). Bleeding manifestations were present in 91.67% of patients, primarily involving the skin and mucous membranes (89.58%), while visceral hemorrhage was rare (5 cases, 2.01%). At the 1-month follow-up, the overall response rate (complete or partial response) reached 99.17%. The long-term (3 years) follow-up revealed a chronic ITP rate of only 8.3%, significantly lower than that in adults. 220 patients (91.67%) received treatment, and treatment selection was age-dependent: the <1 year group primarily received IVIG, whereas the >3 years group more frequently received corticosteroid therapy. Immunological analysis demonstrated an elevated CD8^+^ T cell proportion (*p* = 0.001), a decreased Treg cell proportion (*p* = 0.018), and a reduced CD4^+^/CD8^+^ ratio (*p* = 0.019) in pediatric ITP patients. Cytokine profiling revealed significantly elevated levels of IL-4 (*p* = 0.039) and IL-10 (*p* = 0.025), alongside a marked reduction in IL-35 (*p* = 0.020).

**Conclusion:**

Pediatric ITP demonstrates significant differences from adult ITP in age distribution, sex characteristics, and prognosis. The underlying immune dysregulation involves decreased regulatory T cells, significantly reduced levels of their specific anti-inflammatory cytokine IL-35 and concomitant expansion of CD8^+^ T cells. The defective Treg/IL-35 axis appears to be a important component in ITP pathogenesis, identifying IL-35 as a promising therapeutic target.

## 1 Introduction

Immune thrombocytopenia (ITP) is an acquired autoimmune disease characterized by a reduced platelet count. As the most common hemorrhagic disease in childhood, the clinical manifestations of ITP are heterogeneous, ranging from asymptomatic cases or mild skin or mucosa bleeding to severe, life-threatening bleeding of various organs (1). ITP is classified as newly diagnosed (less than 3 months), persistent (3 to 12 months), or chronic (more than 12 months), with approximately 20% of pediatric patients progressing to chronic phase (2). The prognosis of pediatric ITP is generally favorable. Current first-line treatment regimens, including intravenous immunoglobulin (IVIG) and corticosteroids, are effective in approximately 80% of patients. However, the remaining 20% experience poor outcomes and require second- or third-line treatment, and may eventually progress to chronic ITP, which significantly impairs the quality of life of children and imposes substantial psychological stress and economic burden (3).

The primary pathogenesis of ITP involves immune dysregulation (4). Previous studies emphasized the predominant role of humoral immunity in the development of ITP. However, recent evidence indicates that cellular immune disorder, particularly the imbalance of T lymphocyte subsets and related cytokines, also plays a critical role (5, 6). CD4^+^CD25^+^Foxp3^+^ regulatory T cells (Tregs) are a subset of T lymphocytes with immunosuppressive functions. Multiple studies have documented decreased Tregs in adult ITP (7). T-cell differentiation is strongly influenced by the local immune microenvironment. Cytokines, as key components of the immune milieu, play a critical role in modulating immune responses. Altered cytokine profiles have been documented in acute ITP. IL-35, the only constitutively immunosuppressive member of the IL-12 family, is primarily secreted by activated CD4^+^CD25^+^ Foxp3^+^Tregs. Neutralization of IL-35 abrogates the immunosuppressive activity of Tregs, underscoring its indispensable role in their regulatory function (8). IL-35 has also been implicated in various autoimmune disorders, such as rheumatoid arthritis, systemic lupus erythematosus, and aplastic anemia (9–11). However, its potential involvement in pediatric ITP remains largely unexplored.

The pathogenesis of ITP differs significantly between children and adults in terms of epidemiology, clinical presentation, treatment strategies, and prognosis (12). Currently, most related research focuses on adult populations, while large-scale studies investigating clinical features, cellular immune function, and inflammatory cytokines in pediatric patients remain limited. This study retrospectively analyzes the clinical characteristics and treatment outcomes of 240 children with ITP admitted to our center. Additionally, we examined the subtypes of lymphocytes and cytokine profiles, including CD4^+^CD25^+^Foxp3^+^Tregs and IL-35 levels in 70 pediatric ITP cases in our center between January 2022 and December 2024, to provide further insights into the pathogenesis, diagnosis, and management of pediatric ITP.

## 2 Methods

### 2.1 Study population

The study was approved by the Medical Ethics Committee of Maternal and Child Health Hospital of Hubei Province, Wuhan, China. The diagnosis of ITP referred to the “Recommendations for diagnosis and treatment of primary immune thrombocytopenia in children (2013 Edition)” (13), the “Adapted guideline for the diagnosis and treatment of primary immune thrombocytopenia for Chinese children (2021)” (14), and the “American Society of Hematology 2019 guidelines for immune thrombocytopenia” (15) with the following criteria: 1. At least two complete blood counts showing decreased platelet count, with normal blood cell morphology. 2. Presence of clinical manifestations such as skin petechial, ecchymosis, mucosal or organ bleeding. 3. Absence of splenomegaly. 4. Exclusion of other secondary causes of thrombocytopenia, such as hypoplastic leukemia, aplastic anemia presenting initially with thrombocytopenia, hereditary thrombocytopenia, and thrombocytopenia secondary to other immune diseases, infections or drug factors (13, 15). Clinical data from 240 pediatric ITP patients who were diagnosed and treated at Maternal and Child Health Hospital of Hubei Province, Tongji Medical College, Huazhong University of Science and Technology between January 2015 and January 2022 were collected. These data included general information, bleeding manifestations, initial platelet count, treatment regimens, and response rates. Additionally, 70 children with ITP hospitalized in our institution between January 2022 and December 2024 were enrolled as the ITP group, and 55 healthy, gender- and age matched children undergoing routine physical examinations during the same period were selected as the healthy control group. Lymphocyte subsets (CD3^+^ total T cells, CD3^+^CD4^+^ helper T (Th) cells, CD3^+^CD8^+^ suppressor T (Ts) cells, CD3^-^/CD19^+^ B cells, CD3^-^/CD16^+^CD56^+^ NK cells, CD4^+^CD25^+^ Foxp3^+^Treg cells) and cytokine levels (IL-2, IL-4, IL-6, IL-10, IFN-γ, TNF-α, IL-35) were analyzed and compared between the two groups. All blood samples were collected in heparin anticoagulant tubes prior to any treatment and processed within 2 hours of collection.

### 2.2 Treatment response assessment

The primary treatments for ITP in our center included IVIG, corticosteroids, and recombinant human thrombopoietin (rhTPO). IVIG and corticosteroids were considered as first-line therapies, whereas rhTPO was reserved for second-line use. Second-line therapy was initiated only after confirmation of no response to first-line treatment and following a comprehensive re-evaluation, including bone marrow cell morphology assessment.

Treatment response was classified into five categories: (1) Complete Response (CR): post-treatment platelet count exceeding 100 × 10^9^/L. (2) Response (R): post-treatment platelet count exceeding 30 × 10^9^/L but less than 100 × 10^9^/L, with at least a twofold increase from baseline and no bleeding. (3) Durable Response (DR): maintenance of either CR or R for more than 4 weeks. (4) No Response (NR): post-treatment platelet count remaining below 30 × 10^9^/L, an increase of less than twofold from baseline, or persistent bleeding. (5) Relapse: a decrease in platelet count to <30×10^9^/L, a reduction to less than twofold the baseline value, or recurrence of bleeding symptoms following an initial response.

### 2.3 Detection of peripheral blood lymphocyte subsets

The proportions and absolute counts of lymphocyte subsets were determined using a FACSCalibur flow cytometer (BD Biosciences, USA). BD IMK kit reagents and BD Trucount tubes were employed for lymphocyte subset analysis.

Briefly, each of 10 µL the following antibody --CD3**-**FITC/CD8**-**PE/CD4**-**APC/CD45**-**PerCP and CD3**-**FITC/CD16 and 56-PE/CD19-APC/CD45-PerCP—was added to separate Trucount tubes. Then, 50 µL of anticoagulated whole blood was added to each tube, mixed thoroughly, and incubated for 15 minutes at room temperature in the dark. Subsequently, 450 µL of red blood cell lysis buffer was added, followed by vortex mixing and incubation for 10 minutes in the dark prior to flow cytometric analysis. Lymphocytes were gated based on CD45 expression and side scatter. Cells within the CD3^+^ region were identified as T lymphocytes. CD3^+^CD4^+^ cells were defined as CD4^+^ Th cells, and CD3^+^CD8^+^ cells as CD8^+^ Ts cells. CD3^-^CD19^+^ cells were identified as B lymphocytes. CD3^-^CD16^+^CD56^+^ cells were defined as NK cells. CD4^+^CD25^+^Foxp3^+^ cells were identified as Treg cells.

### 2.4 Cytokine detection

Plasma concentrations of IL-35, IL-2, IL-4, IL-6, IL-10, IFN-γ, and TNF-α were measured using enzyme-linked immunosorbent assay (ELISA). Briefly, 2 mL of peripheral blood was collected in heparin anticoagulant tubes and centrifuged at 500 × g for 10 minutes to isolate plasma. Cytokine concentrations were determined using a double-antibody sandwich method, following the manufacturer’s instructions. The assay procedure was as follows: blank control wells, standard wells, and sample wells were prepared. The blank wells received no sample or enzyme conjugate, while other steps were identical. Standard wells were loaded with 50 µL of standards at various concentrations. For sample wells, 40 µL of sample diluent was added first, followed by 10 µL of the test sample. Subsequently, 100 µL of enzyme conjugate was added to each well, except the blank wells. The plate was sealed and incubated at 37 °C for 60 minutes. The wash buffer (20 × concentrate) was diluted 1:20 with distilled water for use. After incubation, the sealing film was removed, the liquid was discarded, and the plate was blotted dry. Each well was filled with wash buffer, left for 30 seconds, and the buffer was discarded. This process was repeated five times, followed by patting dry. Subsequently, 50 µL of Chromogen Solution A was added to each well, and then 50 µL of Chromogen Solution B were added sequentially to each well. The plate was gently mixed and incubated at 37 °C in the dark for 15 minutes for color development. The reaction was terminated by adding 50 µL of Stop Solution to each well (the blue color immediately turned yellow). The optical density at 450 nm was measured within 15 minutes after adding the stop solution, with the blank well used for zeroing the spectrophotometer.

### 2.5 Statistical analysis

Statistical analysis was performed using SPSS version 22.0. Non-normally distributed continuous data were expressed as median (P25, P75), and the Mann-Whitney U test (for two groups) or the Kruskal-Wallis H test (for multiple groups) was used. Categorical data were expressed as percentages (%) and the Chi-square test or Fisher-Freeman-Halton exact test (for R×C tables or when expected counts were less than 5) was used. The Bonferroni correction was used to adjust *p*-values for multiple pairwise comparisons. No missing data existed for the primary demographic, clinical and laboratory variables analyzed in this study. A *p*-value of less than 0.05 was considered statistically significant.

## 3 Results

### 3.1 Baseline characteristics

A total of 240 children with primary ITP were enrolled, including 142 males and 98 females, with a male-to-female ratio of 1.45:1.00. The median age at onset was 11 months (range, 2 months to 13 years). Patients were categorized into four age groups: <1 year, 1–3 years, 3–6 years, and ≥6 years. The <1 year group included 124 patients (51.67% of the total cohort), with 87 males and 37 females, a male-to-female ratio of 2.35:1. The 1–3 years group included 67 patients with 31 males and 36 females, with a male-to-female ratio of 0.86:1. The 3–6 years group included 36 patients with 18 males and 18 females, a male-to-female ratio of 1:1. The ≥6 years group included 13 patients with 6 males and 7 females, a male-to-female ratio of 0.86:1. The majority of enrolled ITP patients (51.67%) experienced onset before one year of age. The distribution of sex across the four age groups differed significantly (*χ²* = 12.981, Cramer’s V = 0.233, *p* = 0.005). *Post-hoc* multiple comparisons revealed a statistically significant difference in gender distribution specifically between the <1 year group and the 1–3 years group, as detailed in **Table 1**.

**Table 1:** Baseline characteristics of pediatric ITP (n=240)*.

Age group (years)	Gender		Total number (n%)Δ	Effect size	*p-*value
	Male	Female			
<1	87(70.2%)_a_	37(29.8%) _a_	124(51.67%)	0.233	0.005
1-3	31(46.3%)_b_	36(53.7%) _b_	67(27.92%)		
3-6	18(50.0%) _a,b_	18(50.0%) _a,b_	36(15.00%)		
≥6	6(46.2%) _a,b_	7(53.8%) _a,b_	13(5.42%)		
Total numberΔ	142(59.17%)	98(40.83%)	240(100%)		

*Post-hoc pairwise comparisons for Pearson’s chi-square test were performed using the Bonferroni method. Effect size: Cramer’s V. Each subscript letter denotes a subset of categories whose column proportions do not differ significantly from each other at the 0.05 level. Δ The denominator for this percentage is the total ITP cohort. Percentages in other rows use the respective age group total as the denominator.

### 3.2 Presence of potential precipitating factors

Among the 240 patients, 88 (36.67%) had potential precipitating factors 1–4 weeks prior to onset, while 152 (63.33%) had no identifiable triggers. These potential precipitating factors included respiratory infections in 59 cases (24.58%) and vaccination in 29 cases (12.08%). The prevalence of a preceding respiratory infection as potential antecedents differed significantly across the four age groups (*χ²* = 32.256, Cramer’s V = 0.367, *p* < 0.001), with the highest proportion in the 3–6 years age group (61.11%). Fisher-Freeman-Halton exact test indicated that the presence of a vaccination history as potential antecedents differed significantly across the four age groups (*χ²* = 11.871, Cramer’s V = 0.22, *p* = 0.005), with the highest proportion in the <1 year age group (19.35%). The analysis of potential precipitating factors and their proportions in children with ITP prior to onset is detailed in **Table 2**.

**Table 2:** Distribution of potential antecedents among pediatric ITP by age group (n=240).

Age group (years)	Identified antecedents no/yes	Infections	Vaccines
<1	81/43 (65.32/34.67%)	19 (15.32%) _a_	24 (19.35%) _a_
1-3	48/19 (71.64/28.37%)	16 (23.89%) _a_	3 (4.48%) _b_
3-6	12/24 (33.33/66.67%)	22 (61.11%) _b_	2 (5.56%) _a,b_
≥6	11/2(84.62/15.38%)	2(15.38%) _a,b_	0(0%) _a,b_
Total	152/88(63.33/36.67%)	59(24.58%)	29(12.08%)
Effect size		0.367	0.22
*p*-value		<0.001*	0.005△

*Post-hoc pairwise comparisons for the Pearson chi-square test were conducted using the Bonferroni correction. Effect size: Cramer’s V.

△: Fisher-Freeman-Halton exact test was applied. Subscript letters following *post-hoc* comparisons indicate subsets of categories whose column proportions do not differ significantly from each other at the α = 0.05 level. Effect size: Cramer’s V.

### 3.3 Initial platelet count

The initial platelet count was 13 (6, 17) × 10^9^/L for male patients and 17 (9, 29.75) × 10^9^/L for female patients. The difference was not statistically significant (*Z* = -1.429, *r* = 0.092, *p* = 0.153), indicating comparable median platelet counts at initial diagnosis between males and females. Patients were categorized into three groups based on their initial platelet count: <20 × 10^9^/L, 20-50 × 10^9^/L and >50 × 10^9^/L. The <20 × 10^9^/L group included 148 patients (61.67%), with 92 males and 56 females. The 20-50 × 10^9^/L group included 86 patients (35.83%), with 50 males and 36 females. The >50 × 10^9^/L group included 6 patients (2.5%), with 3 males and 3 females. Fisher-Freeman-Halton exact test revealed no statistically significant difference in the sex distribution across these platelet count groups (*χ²* = 0.72, Cramer’s V = 0.055*, p* = 0.697), as detailed in **Table 3**.

**Table 3:** Comparison of initial platelet counts by patient sex (n=240).

Platelet parameters	Male (n=142)	Female (n=98)		Effect size	*p*-value
Platelet counts(×10^9^/L) [M (P25, P75)]	13 (6, 24)	17 (9, 29.75)		0.092	0.153 *
Sex Distribution across Platelet Count, [n (%)]					
<20 × 10^9^/L	92 (62.2%)	56 (37.8%)			0.697△
20~50 ×10^9^/L	50 (58.1%)	36 (41.9%)	0.055		
>50 ×10^9^/L	3 (50.0%)	3 (50.0%)			

*Mann-Whitney U test was applied. Effect size: *r*.

△ Fisher-Freeman-Halton exact test was applied. Effect size: Cramer’s V.

### 3.4 Bleeding manifestations

Of the 240 children, 20 cases (8.3%) presented without bleeding manifestations, exhibiting a median platelet count of 29.50 (15.75, 36.75) × 10^9^/L. The remaining 220 cases (91.67%) exhibited bleeding manifestations, with a significantly lower median platelet count of 13.00 (6.00, 25.00) × 10^9^/L. Among those with bleeding, 215 (89.58%) had superficial bleeding manifestations including skin or mucosal bleeding, gingival bleeding or epistaxis, with a median platelet count of 13.00 (6.00, 25.50)×10^9^/L. 5 cases (2.08%) had concomitant organ hemorrhage, including 3 cases of gastrointestinal bleeding, 1 case of urinary tract bleeding and 1 case of intracranial hemorrhage. This subgroup had a median platelet count of 8.00 (4.50, 18.00) × 10^9^/L. The median platelet count was significantly higher in patients without bleeding manifestations compared to those with any bleeding (*Z* = -2.96, *r* = 0.191, *p* = 0.003). Although patients with visceral hemorrhage had a lower median platelet counts than those with superficial bleeding, the difference was not statistically significant (*Z* = -0.876, *r* = 0.057, *p* = 0.381) (**Table 4**).

**Table 4:** Comparison of initial platelet counts by bleeding manifestation (n=240)*.

Bleeding manifestations	Platelet counts (×10^9^/L) [M (P25, P75)]	Effect size	*p*-value
no bleeding (20)	29.50 (15.75, 36.75)	0.191	0.003
with bleeding manifestations (220)	13.00 (6.00, 25.00)		
Superficial Bleeding (petechiae, epistaxis, gingival) (215)	13.00 (6.00, 25.00)	0.057	0.381
Visceral Hemorrhage Gastrointestinal (3) urinary tract bleeding (1) Intracranial hemorrhage (1)	8.00 (4.50, 18.00)		

*Mann-Whitney U test was applied. Effect size: *r*.

### 3.5 Diagnostic classification

All 240 enrolled patients were followed for 3 years. Of these, 220 (91.67%) were classified as newly diagnosed ITP, distributed as follows: <1 year (n=124), 1–3 years (n=53), 3–6 years (n=33), and ≥6 years (n=10). The remaining 20 cases (8.33%) were classified as either persistent or chronic ITP, distributed as: <1 year (n=0), 1–3 years (n=14), 3–6 years (n=3), and ≥6 years (n=3). The proportion of persistent or chronic ITP varied significantly across the age groups (*χ²* = 31.948, Cramer’s V = 0.365, *p* < 0.001). Notably, all patients in the <1 year group were diagnosed as newly diagnosed ITP. *Post-hoc* multiple comparisons revealed that the proportion with disease duration ≥3 months in this group (0%) was significantly lower than that in all other age groups. In contrast, no statistically significant differences were observed among the other age groups. Details are presented in **Table 5**.

**Table 5:** Comparison of proportions of persistent/chronic ITP across age Groups (n, %)*.

Age group (years)	Newly diagnosed ITP	Persistent/chronic ITP	Effect size	*p*-value
<1	124(100.0%)_a_	0(0.0%)_a_	0.365	<0.001
1-3	53(79.1%)_b_	14(20.9%)_b_		
3-6	33(91.7%)_b_	3(8.3%)_b_		
≥6	10(76.9%)_b_	3(23.1%)_b_		
Total	220(91.7%)	20(8.3%)		

*Fisher-Freeman-Halton exact test was applied. Effect size: Cramer’s V. Subscript letters following *post-hoc* comparisons indicate subsets of categories whose column proportions do not differ significantly from each other at the α = 0.05 level.

### 3.6 Treatment profiles

Patients were categorized based on initial therapy: (1) No therapy group. (2) IVIG monotherapy: 0.8–1 g/kg per administration, for 1 to 2 days. (3) Corticosteroid monotherapy: corticosteroid include methylprednisolone, dexamethasone and prednisone. (4) Combined IVIG and corticosteroid therapy. 220 patients (91.67%) received treatment. The proportion of patients receiving treatment differed significantly among age groups (*χ²* = 10.105, Cramer’s V = 0.205, *p* = 0.011). *Post-hoc* multiple comparisons indicated that the <1 year group received treatment significantly more often than the 1–3 years group, but did not differ significantly from the 3–6 years or ≥6 years groups. The distribution of treated versus untreated patients across age groups are detailed in **Table 6**.

**Table 6:** Comparison of treatment rates across age groups in pediatric ITP*.

Age group (years)	Treatment or not		Total	Effect size	*p*-value
	no	yes			
<1	5(4.0%)_a_	119(96%)_a_	124(100.00%)	0.205	0.011
1-3	12(17.91%)_b_	55(82.09%)_b_	67(100.00%)		
3-6	2(5.56%)_ab_	34(94.44%)_ab_	36(100.00%)		
≥6	1(7.69%)_ab_	12(92.31%)_ab_	13(100.00%)		
Total	20(8.33%)	220(91.67%)	240(100%)		

*Fisher-Freeman-Halton exact test was applied. Effect size: Cramer’s V. Subscript letters following *post-hoc* comparisons indicate subsets of categories whose column proportions do not differ significantly from each other at the α = 0.05 level.

Among the 220 treated patients, 147 (66.82%) received IVIG monotherapy, 21 (9.55%) received corticosteroid pulse monotherapy, and 52 (23.64%) received combination therapy. Further analysis of the initial treatment choice within each age group revealed a statistically significant difference in the proportion of patients for whom IVIG was the preferred initial regimen (*χ²* = 9.758, Cramer’s V = 0.15, *p* = 0.02). Pairwise comparisons identified the most significant difference between the <1 year and 3–6 years age groups (*p* = 0.006), suggesting a stronger preference for IVIG monotherapy in <1 year group.

The preference for corticosteroids as the initial regimen also varied significantly among different age groups (*χ²* = 23.27, Cramer’s V = 0.23, *p* < 0.001). Pairwise comparisons showed significant differences between the <1 year group and each of the three older age groups, indicating that corticosteroid use as first-line therapy increased with age. In contrast, the proportion receiving combination therapy did not differ significantly across the age groups (*χ²* = 4.058, Cramer’s V = 0.1, *p* = 0.258). When patients were grouped by the initial treatment received, there was no statistically significant difference in age distribution across the three initial treatment strategies (*H* = 5.365, *η*²= 0.025, *p* = 0.068),. *Post-hoc* pairwise comparisons revealed that children receiving corticosteroids were significantly older than those treated with IVIG (*Z* = 1.980, *r* = 0.153, *p* = 0.048), consistent with age-stratified findings. Details are provided in **Table 7**.

**Table 7:** Comparison of initial treatment strategies and age in pediatric ITP.

Age group (years)	IVIG (n,%)	corticosteroids(n,%)	combination therapy(n,%)	Total(n)
<1	87(73.11%)_a_	2(1.68%) _a_	30(25.21%) _a_	119
1-3	37(67.27%)_ab_	8(14.55%) _b_	10 (18.18%) _a_	55
3-6	16(47.05%)_b_	7(20.59%) _b_	11 (32.35%) _a_	34
≥6	7(58.33%)_ab_	4(33.33%) _b_	1 (8.33%) _a_	12
Total	147(66.82%)	21(9.55%)	52(23.64%)	220
Effect size	0.15	0.23	0.1	
*p*-value	0.02*	<0.001*	0.258*	
Age (month) [M (P25, P75)]	6.28 (3.75, 17.75)	27.5 (12.00, 54.75)	10.03 (5.07, 28)	
Effect size	0.025	0.153		
*p*-value	0.068△	0.048□		

*Fisher-Freeman-Halton exact test was applied. Effect size: Cramer’s V. Subscript letters following *post-hoc* comparisons indicate subsets of categories whose column proportions do not differ significantly from each other at the α = 0.05 level.

△ Kruskal-Wallis H test was applied for the comparison of age among 3 treatment groups. Effect size: *η²*.

□ Mann-Whitney U test was applied for the comparison of age between IVIG and corticosteroids. Effect size: *r*.

Among the 240 patients, only 4 (1.67%) required second-line therapy (primarily rhTPO at our center). The remaining majority (98.33%) achieved favorable outcomes with first-line treatments. A comparison of initial platelet counts was performed among patients managed with different treatment strategies. The results demonstrated that the median platelet count in the untreated group was 38.00 (29.50, 53.50) × 10^9^/L, significantly higher than the count of 13.00 (6.00, 24.5) ×10^9^/L in the treated group, with the difference being statistically significant (*Z* = 4.278, *r* = 0.276, *p* < 0.001). Furthermore, among the treated patients, the initial platelet counts were analyzed based on the specific first-line therapy received. The median platelet count was 15.00 (8.50, 25.00) × 10^9^/L for those who received IVIG as initial therapy, 13.00 (6.75, 24.25) × 10^9^/L for those starting with corticosteroids, and 10.50 (5.00, 22.75) × 10^9^/L for those initiated on combination of IVIG and corticosteroid therapy. However, the differences in initial platelet counts among these three treatment subgroups were not statistically significant (*H* = 3.01, *η*² = 0.0047, *p* = 0.22). Detailed data are presented in **Table 8**.

**Table 8:** Comparison of initial platelet counts among patients managed with different treatment strategies (n=240).

Treatment strategy	Patient number n(%)	Platelet count(×10^9^/L)	Effect size	*p*-value
No treatment	20(8.33%)	38.00(29.50, 53.50)	0.276	<0.001*
With treatment	220(91.67%)	13.00(6.00, 24.5)		
IVIG	147(61.25%)	15.00(8.50, 25.00)	0.0047	0.22^△^
corticosteroids	21(8.75%)	13.00(6.75, 24.25)		
combination therapy	52(21.67%)	10.50(5.00, 22.75)		
Total	240(100%)	26(16, 36)		

*Mann-Whitney U test was applied, effect size: *Z*. ^△^Kruskal-Wallis H test was applied, effect size: *η*².

### 3.7 Treatment response

Patients were categorized into three groups based on platelet count at one month after initial treatment: CR (platelet count ≥100 × 10^9^/L), R (platelet count 30-100 × 10^9^/L) and NR (platelet count <30 × 10^9^/L). The results indicated that 200 patients (83.33%) maintained CR, with a platelet count of 266 (165.5, 361.25) × 10^9^/L. 38 patients (15.83%) were in the R group, with a platelet count of 61 (46, 89) × 10^9^/L, while 2 patients (0.83%) showed no response, with platelet count of 19 × 10^9^/L and 25 × 10^9^/L, respectively.

A comparison of the initial platelet count among these three response groups revealed no statistically significant difference (*Z* = 0.172, *r* = 0.011, *p* = 0.863), suggesting that the treatment response at one month was not associated with the initial platelet count. Detailed results are presented in **Table 9**.

**Table 9:** Treatment response at one month and its association with initial platelet count.

Treatment response	n(%)	Platelet count at 1 Month post-treatment (×10^9^/L)	Initial platelet count (×10^9^/L)	Effect size	*p*-value
NR	2(0.83%)	25,19	8,10	0.011	0.863
R	38(15.83%)	61(46,89)	32(11,36)		
CR	200(83.33%)	266(165.5,361.25)	13(6,24)		
Total	240(100%)	205(132, 321)	26(16,36)		

*Mann-Whitney U test was applied. Effect size: *r*.

### 3.8 Alterations in cellular immune function in ITP patients

Lymphocyte subset profiles in newly diagnosed ITP children were analyzed to elucidate their cellular immune status. The results revealed significant differences in CD3^+^ T cell count, CD3^+^CD8^+^ T cell count and percentages, Treg percentage, and the CD4^+^/CD8^+^ ratio between the ITP and healthy control group.

Specifically, the total CD3^+^ T cell count was significantly elevated in the ITP group compared to the healthy controls: 3072.44 (1955.89, 4295.42)/µL vs. 2276.61 (1228.90, 3052.70)/µL (Z = -2.498, r = 0.22, *p* = 0.012). Similarly, both the absolute count and the percentage of CD3^+^CD8^+^ T cells were significantly higher in the ITP group: the absolute count was 1037.43 (732.10, 1605.72)/µL vs. 716.30 (335.45, 1046.04)/µL (*Z* = -3.296, *r* = 0.30, *p* = 0.001), and the percentage was 25.78% (20.36%, 30.14%) vs. 21.16% (17.45%, 27.32%) (*Z* = -2.628, *r* = 0.24, *p* = 0.009). Conversely, the CD4^+^/CD8^+^ ratio was significantly lower in the ITP group: 1.41 (1.10, 1.99) vs. 1.80 (1.41, 2.32) (*Z* = -2.347, *r* = 0.21, *p* = 0.019). The percentage of Tregs was significantly elevated in the ITP group compared to the healthy controls: 4.01 (2.68, 5.96) % vs. 5.83(3.92, 6.85) % (*Z* = -2.389, *r* = 0.21, *p* = 0.018). All the aforementioned differences were statistically significant. Detailed data are presented in **Table 10**. Then the patients were divided into <3 years and ≥3 years group. After age stratification, the CD8^+^ T cell elevation and Treg reduction was predominantly observed in children in <3 years group, whereas no significant differences were detected in ≥3 years group. (**Supplementary Table 1**).

**Table 10:** Comparison of lymphocyte subsets between newly diagnosed pediatric ITP patients and healthy controls*.

Lymphocyte subsets	ITP group	Healthy control group	Effect size	*p*-value
CD3^+^ (/L)	3072.44(1955.89, 4295.42)	2276.61(1228.90, 3052.70)	0.22	0.012
CD3^+^CD8^+^ (/L)	1037.43(732.10, 1605.72)	716.30(335.45, 1046.04)	0.30	0.001
CD4^+^/CD8^+^ ratio	1.41(1.1, 1.99)	1.80(1.41, 2.32)	0.21	0.019
CD3^+^CD8^+^(%)	25.78(20.36, 30.14)	21.16(17.45, 27.32)	0.24	0.009
Treg (%)	4.01(2.68, 5.96)	5.83(3.92, 6.85)	0.21	0.018

*Mann-Whitney U test was applied. Effect size: *r*.

### 3.9 Cytokine profile alterations in pediatric ITP

Serum levels of cytokines IL-2, IL-4, IL-6, IL-10, IFN-γ, TNF-α, and IL-35 were analyzed in newly diagnosed ITP children to characterize associated cytokine changes. Significant differences in concentrations of IL-4, IL-10, and IL-35 were observed between the ITP and healthy control group.

Specifically, IL-4 levels were significantly higher in the ITP group compared to healthy controls: 3.10 (1.64, 4.30) pg/mL vs. 1.63 (0.96, 3.78) pg/mL (*Z* = -2.069, *r* = 0.19, *p* = 0.039). Similarly, IL-10 levels were elevated in ITP patients: 6.45 (3.88, 10.57) pg/mL vs. 4.88 (2.39, 7.81) pg/mL (*Z* = -2.240, *r* = 0.20, *p* = 0.025). In contrast, IL-35 levels were significantly lower in the ITP group: 146.41 (21.45, 320.48) pg/mL vs. 438.98 (202.64, 1288.60) pg/mL (*Z* = -2.318, *r* = 0.21, *p* = 0.020). All these differences were statistically significant (**Table 11**). No statistically significant differences were observed between the two groups in the concentrations of IL-2, IL-6, IFN-γ and TNF-α. After age stratification, the above alterations were predominantly observed in children ≥3 years group, while no significant differences were detected in <3 years group. (**Supplementary Table 2**).

**Table 11:** Comparison of cytokine profiles between newly diagnosed pediatric ITP patients and healthy controls*.

Cytokine	ITP group (n=70)	Healthy control group (n=55)	Effect size	*p*-value
IL-2 (pg/mL)	2.445(1.41, 5.45)	2.02(1.23, 4.66)	0.06	0.473
IL-4 (pg/mL)	3.10(1.64, 4.30)	1.63(0.96, 3.78)	0.19	0.039
IL-6 (pg/mL)	11.05 (4.32, 23.99)	14.13 (5.48, 32.13)	-0.08	0.393
IL-10 (pg/mL)	6.45(3.88, 10.57)	4.88(2.39, 7.81)	0.20	0.025
IL-35 (pg/mL)	146.41(21.45, 320.48)	438.98(202.64, 1288.6)	0.21	0.020
IFN-γ (pg/mL)	2.77(1.81,3.67)	2.57(1.69, 6.3)	0.01	0.881
TNF-α (pg/mL)	4.26(2.44, 12.41)	2.81(1.26, 12.96)	0.14	0.131

*Mann-Whitney U test was applied. Effect size: *r*.

## 4 Discussion

The incidence of ITP demonstrates distinct variations according to age and sex (17). Whereas older adults and young women exhibit higher incidence rates, the disease is less common in children than in adults (18–20). This study analyzed the clinical characteristics of 240 newly diagnosed pediatric ITP patients, revealing distinct features in clinical presentation, treatment response, and immunological profiles.

Our findings revealed that children in the <1 year age group constituted the largest subgroup, accounting for 51.67% of the 240 ITP cases, a proportion significantly higher than that in other age groups. This may be related to the immature immune system in this age group, predisposing them to various viral infections and vaccine responses. Previous research indicated that adult ITP is more prevalent in females than males (17). In contrast, our study indicated a significant male predominance only in the <1 year age group, with no significant sex differences observed in other pediatric age groups. This suggests a discrepancy in sex distribution between childhood and adult ITP. Such a discrepancy may stem from the higher prevalence of autoimmune diseases in adult women, which can affect the hematopoietic system, with immune thrombocytopenia sometimes serving as the sole initial manifestation (21). Conversely, the lower prevalence of autoimmune diseases in children may explain the absence of female predominance. This differential pattern implies distinct pathogenic mechanisms between childhood and adult ITP, particularly in infantile cases. As reported by Monto, male infants exhibit greater susceptibility to respiratory infections, which may partially explain this sex bias, although other potential predisposing factors warrant further investigation (22).

ITP is associated with various triggers of immune dysregulation, with viral infections representing the most significant etiology, while a smaller proportion of cases follow vaccination (7, 23). The underlying mechanism primarily involves molecular mimicry, which triggers autoimmune responses against platelets (16). Our study demonstrated that 29 patients (12.08%) had a vaccination history within 1–4 weeks prior to disease onset, predominantly in the <1 year group (24 cases), involving vaccines such as measles, hepatitis B, and pentavalent vaccines. Additionally, 59 patients (24.58%) had recent respiratory infections, most frequently in the 3–6 years group (22 cases). In China, children under one year of age receive multiple scheduled vaccinations, whereas children over three years old begin collective life in kindergarten with increased outdoor activities, resulting in a higher frequency of respiratory infections. These factors align with the peak age groups for ITP onset identified in our cohort. However, although a temporal relationship was observed between vaccination or respiratory infection and ITP onset, causality cannot be established based on these data. These events may act as potential triggers in susceptible individuals, but further mechanistic studies are required to confirm a causative link.

Our findings indicated no statistically significant difference in the median initial platelet count between males and females. Bleeding was the primary clinical manifestation of ITP, observed in 220 cases (91.67%). Superficial bleeding involving the skin and mucosa was most common, occurring in 215 cases (89.58%), while visceral hemorrhage was rare, occurring in only 5 cases (2.01%). Compared with adults, pediatric ITP patients demonstrate higher overall bleeding rates but fewer severe bleeding events, potentially due to comorbidities and the use of antiplatelet medications (e.g., anti-inflammatory drugs, antiplatelet agents) in adults (20). Our data demonstrated that the median platelet count was significantly higher in patients without bleeding manifestations than in those with bleeding (29.50 vs. 13.00 ×10^9^/L, *p* < 0.05). However, no significant difference in platelet count was observed between the visceral and superficial bleeding groups, suggesting that although the degree of thrombocytopenia correlates with overall bleeding risk, it does not predict an increased risk of visceral hemorrhage.

Short-term follow-up (1 month) demonstrated an overall response rate of 99.17%, comprising 83.33% CR and 15.83% R, with only 0.83% showing NR. These results are significantly superior to response rates reported in adult ITP. Long-term follow-up (3 years) revealed that most pediatric ITP cases were newly diagnosed, with the proportions of persistent and chronic ITP increasing with age. However, the overall chronic ITP rate (8.3%) remained substantially lower than in adults (approximately 75%), confirming a more favorable prognosis in children (24). Chronic ITP was predominantly concentrated in the ≥6 years group, while no persistent cases were identified in the <1 year group. The remarkably high one-month overall response rate of 99.17% observed in our pediatric ITP cohort warrants further consideration. This can be attributed to both the timely initiation of first-line therapy and the favorable natural history of childhood ITP. Although patients received different first-line therapies based on age, bleeding severity, parental preference, and economic considerations, all subgroups demonstrated excellent short−term outcomes. In addition, the natural history of pediatric ITP differs fundamentally from that of adult disease; children are more likely to experience acute, self−limited thrombocytopenia with spontaneous remission. Furthermore, the one−month follow−up window, while sufficient to assess initial treatment response, captures the peak of therapeutic effect and may not reflect long−term disease persistence. Indeed, our three−year follow−up data showed that 8.3% of patients progressed to chronic ITP, supporting the notion that short−term remission does not entirely preclude the subsequent development of chronic disease. Therefore, longer follow−up remains essential to identify patients at risk for chronic ITP. Nevertheless, both the high early response at one month and the low three-year chronicity rate indicate that pediatric ITP generally carries a favorable prognosis despite heterogeneous treatment approaches. Notably, no patients with a vaccination history progressed to chronic ITP, suggesting that age and recent vaccination history may serve as effective prognostic indicators.

Current pediatric ITP guidelines recommend IVIG and corticosteroids as first-line therapies (15). Their mechanisms differ: IVIG primarily acts by blocking platelet antibody-mediated clearance, whereas corticosteroids suppress anti-platelet antibody production. Both regimens are weight-based but differ in adverse effect profiles and cost. In our study, the majority of patients (91.67%) received first-line treatment, with the highest treatment rate in the <1 year group, potentially due to the immature immune system of infants, a greater propensity for disease progression, and possibly stronger parental inclination toward intervention. Treated patients had significantly lower initial platelet counts than untreated counterparts (13.00 vs. 38.00×10^9^/L), indicating that treatment decisions closely correlate with initial platelet counts at presentation. A distinct age-based preference in the choice of first-line therapy was observed: the <1 year group predominantly received IVIG monotherapy, maybe considering lower growth impact concerns and manageable cost associated with lower body weight, whereas the >3 year group more frequently received corticosteroids, considering economic factors and relatively controllable adverse effect risks. The lack of correlation between initial platelet counts and treatment selection suggests that the choice of the initial therapeutic agent in our cohort was a complex decision influenced by a comprehensive consideration of multiple factors, including efficacy, parental preference, and financial circumstances, rather than the presenting platelet count alone. Only 4 patients (1.67%) required second-line therapy (rhTPO), and these patients had relatively higher initial platelet counts with subtle symptoms. We hypothesize that their less pronounced thrombocytopenia and milder bleeding manifestations may have led to a more insidious disease onset and delayed recognition, potentially contributing to a more protracted clinical course.

Traditional views attribute platelet destruction in ITP primarily to autoantibodies targeting platelet glycoproteins (GPIIb/IIIa and GPIb/IX). However, recent studies suggest that these antibodies can be detected in only approximately 53% of patients, indicating additional pathogenic mechanisms beyond humoral immunity. T-cell dysfunction plays a crucial role in ITP pathogenesis (25, 26).

Our study and numerous previous investigations confirm immune imbalance in ITP patients, characterized by cytotoxic T-cell proliferation and reduced Treg numbers (27). Olsson et al. demonstrated that stimulated CD8^+^ T cells can directly lyse autologous platelets and megakaryocytes or secrete pro-inflammatory cytokines such as IFN-γ, and activating macrophage phagocytosis, thereby contributing to platelet destruction (28). Notably, Amna et al. identified a distinct subset of terminally differentiated CD8^+^ T cells (CD45RA^+^CD62L^−^) that expands during thrombocytopenia, forms aggregates with autologous platelets and releases IFN-γ, triggering platelet activation and apoptosis via TCR-mediated cytotoxic granule release (5). This phenomenon is particularly prominent in refractory ITP, further substantiating the involvement of specific CD8^+^ T-cell subsets in ITP pathogenesis.

Concurrently, reduced Treg numbers exacerbate immune dysregulation. Tregs suppress the activation of CD8^+^ T cells and antibody production through cell-contact-dependent mechanisms and the secretion of inhibitory cytokines, such as IL-10 and TGF-β. Our observed reduction of Tregs is consistent with previous findings by Zahran, Elsayh and Stasi (29, 30). Importantly, Treg reduction and CD8^+^ T-cell expansion are not isolated phenomena; they may influence each other and create a “vicious cycle”: activated CD8^+^ T cells and their secreted pro-inflammatory cytokines (e.g., IFN-γ) can inhibit Treg differentiation or function, while Treg deficiency subsequently removes suppression of CD8^+^ T cells, promoting their further activation and expansion. Our study demonstrated a decreased CD4^+^/CD8^+^ ratio in pediatric ITP, indicating impaired cellular immune tolerance and aberrant autoimmune activation associated with both elevated CD8^+^ T cells and reduced Tregs (CD4^+^). Although some studies have reported an increased CD4^+^/CD8^+^ ratio in ITP (31), contradicting our findings, such discrepancies may be attributed to variations in study populations and disease staging.

Cytokine network imbalance plays a pivotal role in ITP pathogenesis. We identified a marked Th2 immune shift, characterized by significantly elevated IL-4 and IL-10 levels. The increased IL-4 level aligns with Talaat et al.’s findings, peaking during the acute disease phase (31). As a crucial Th2 cytokine, IL-4 promotes B-cell differentiation and antibody class switching, playing a key role in anti-platelet antibody production. Webber et al. further confirmed the significance of IL-4 in autoantibody generation (32). Concurrently, significantly elevated IL-10 levels demonstrate dual effects. Although traditionally considered anti-inflammatory, studies by Del Vecchio and Zhou indicate its immunostimulatory effects on B cells (33, 34). This elevation may represent a compensatory immunoregulatory response that remains insufficient to restore immune tolerance amid overall immune imbalance.

Notably, we found significantly reduced IL-35 levels in pediatric ITP patients. Studies demonstrate that neutralizing IL-35 abrogates Treg-mediated immunosuppression, indicating inseparable functional links between IL-35 and Treg activity (8, 35). Multiple studies implicate IL-35 in various autoimmune diseases including Guillain-Barré syndrome (36), rheumatoid arthritis (37), and systemic lupus erythematosus (10). Combined with the observed reduction of Tregs and IL-35 in our pediatric ITP cohort, we reasonably speculate that decreased serum IL-35 levels may impair Treg immunosuppressive activity, potentially disrupting the Treg/Th17 balance during the development of childhood ITP. Unlike Yang’s study, we found no correlation between serum IL-35 levels and platelet count, possibly due to differences in patient populations (38). Our cohort primarily comprised newly diagnosed ITP cases, whereas Yang’s study focused mainly on chronic ITP patients. Interestingly, although previous studies report elevated IL-12 and IL-23 in ITP patients (39), we observed reduced IL-35 in newly diagnosed pediatric ITP, suggesting that imbalance among the IL-12 family cytokines may represent a characteristic cytokine profile in ITP, providing novel perspectives for understanding its immunopathogenesis. Research indicates that Treg-derived IL-35 promotes iTr35 generation and increases the proportions of nTreg, thereby ameliorating inflammation (40). Given that IL-35 can potently generate iTr cells *in vitro* and maintain effective regulatory functions *in vivo* (41), it emerges as a promising therapeutic target for further investigation, particularly for chronic and refractory ITP.

It is noteworthy that age-stratified analysis revealed a complete dissociation of these immunological abnormalities: the CD8^+^ T cell elevation was confined to children in <3 years age group, whereas the Th2 cytokine (IL-4 and IL-10) elevation and Treg/IL-35 deficiency were observed predominantly in those aged ≥3 years. This suggests an age-dependent shift in the immunopathogenesis of pediatric ITP (42, 43). In early childhood (<3 years), the disease appears to be predominantly driven by T cell-mediated cellular immunity, characterized by CD8^+^ T cell expansion and disrupted CD4^+^/CD8^+^ homeostasis (29). This may be attributable to the relative immaturity of regulatory networks during this developmental window (44), rendering young children more susceptible to uncontrolled cytotoxic T cell responses upon immune triggers. Conversely, in older children (≥3 years), the immune dysregulation shifts toward a Th2-B cell axis, with elevated IL-4 and IL-10 promoting humoral autoimmunity, accompanied by a pronounced Treg/IL-35 axis defect that underlies impaired immune tolerance (45). The confinement of Treg/IL-35 deficiency to the older age group suggests that regulatory dysfunction may become functionally relevant mainly after immune system maturation. These findings highlight the heterogeneity of immune mechanisms across different ages in pediatric ITP and underscore the critical importance of age stratification in both mechanistic studies and therapeutic strategies. Understanding this age-dependent immunological transition may facilitate more targeted, age-appropriate interventions for children with ITP.

This study has several limitations. First, given its observational design, the role of the Treg/IL-35 axis in ITP pathogenesis should be considered hypothesis-generating and requires further validation in functional experimental models. Second, the modest sample size of patients aged 3 years or older may have limited the power to reflect the true statistic situation. Third, the cellular sources of cytokines remain unclarified, serial pre- and post-treatment samples for dynamic immune monitoring are unavailable, and mechanistic insights are insufficient. Future studies with larger, age-balanced cohorts, combined with single-cell sequencing, longitudinal monitoring, and gene-editing technologies, are warranted to further elucidate the immunological patterns and mechanisms of pediatric ITP.

## 5 Conclusion

This study elucidates a potential important mechanism of immune dysregulation in pediatric ITP. We identified a critical immunoregulatory defect characterized by a significant reduction in both the Treg population and IL-35, a potent anti-inflammatory cytokine specifically secreted by Tregs. This deficiency was concomitant with the activation and expansion of CD8^+^ T cells, leading to a decreased CD4^+^/CD8^+^ ratio. Our findings indicate that Treg dysfunction involves not only numerical deficiency but also loss of its key effector molecule, IL-35, which fundamentally compromises immune suppression. In this context, the compensatory elevation of IL-10 appears insufficient to counteract the autoimmune attack triggered by the defective Treg/IL-35 axis. Consequently, disruption of the Treg/IL-35 balance may represent a potential important mechanism in the pathogenesis of pediatric ITP. Combined assessment of these parameters may serve as a novel strategy for evaluating immune status and disease prognosis. Restoring Treg function and IL-35 levels thus represents a promising therapeutic approach for immune reconstitution in ITP.

## Data Availability

The original contributions presented in the study are included in the article/**Supplementary Material**. Further inquiries can be directed to the corresponding author/s.
