# Supplementary material for: Clinical characteristics and decreased CD4+CD25+Foxp3+ regulatory T cells and IL-35 in pediatric immune thrombocytopenia in a single center
Source: Front Immunol. 2026 Mar 10;17:1782560. doi: 10.3389/fimmu.2026.1782560 (PMC13011169; doi:10.3389/fimmu.2026.1782560)
Supplement: Supplementary file 1 [file Table1.doc]

Supplementary Table 1. Comparison of lymphocyte subsets between newly diagnosed pediatric ITP patients and healthy controls in different age groups*

| lymphocyte  subsets | ＜ 3 years group | | |  | ≥ 3 years group | | |
| --- | --- | --- | --- | --- | --- | --- | --- |
|  | ITP  (n=52 ) | healthy control  (n= 42) | *Z* / *r* / *p*-value |  | ITP  (n=18 ) | healthy control  (n= 13) | *Z* / *r* / *p*-value |
| CD3^+^ (/L) | 3511.23(2578.58, 4910.08) | 2543.85(1308.44, 4214.29) | -2.198  /0.227  /0.028 |  | 1973.39 (1334.56, 2644.59) | 1705.33 (1092.91, 3034.23) | -0.560  /0.100  /0.576 |
| CD3^+^CD8^+^ (/L) | 1194.9 (860.46, 1951.41) | 801.86 (288.87, 1462.07) | -2.466  /0.254  /0.014 |  | 755.07 (509.92, 1035.38) | 685.72 (339.54, 769.97) | -1.452  /0.261  /0.146 |
| CD4^+^/CD8^+^  ratio | 1.44(1.14, 2.09) | 1.83 (1.43, 3.00) | -2.21  /0.228  /0.027 |  | 1.28 (1.04, 1.73) | 1.78 (0.96, 2.19) | -1.347  /0.243  /0.178 |
| CD3^+^CD8^+^ (%) | 25.37(19.76, 29.03) | 20.77 (16.03, 22.60) | -2.504  /0.258  /0.012 |  | 26.89 (21.45, 32.87) | 21.33 (19.32, 27.57) | -1.688  /0.300  /0.091 |
| Treg (%) | 4.33(2.98, 9.17) | 5.20(2.00, 6.59) | -1.513  /0.16  /0.130 |  | 3.46(1. 20, 5.58) | 7.25(3.59, 12.10) | -1.990/  /0.361  /0.047 |

* Mann-Whitney U test was applied. Effect size: *r*.
