# Supplementary material for: Clinical characteristics and decreased CD4+CD25+Foxp3+ regulatory T cells and IL-35 in pediatric immune thrombocytopenia in a single center
Source: Front Immunol. 2026 Mar 10;17:1782560. doi: 10.3389/fimmu.2026.1782560 (PMC13011169; doi:10.3389/fimmu.2026.1782560)
Supplement: Supplementary file 2 [file Table2.doc]

Supplementary Table 2. Comparison of cytokine profiles between newly diagnosed pediatric ITP patients and healthy controls in different age groups *

| cytokine  (pg/mL) | ＜ 3 years group | | |  | ≥ 3 years group | | |
| --- | --- | --- | --- | --- | --- | --- | --- |
|  | ITP | healthy control | *Z* / *r* / *p*-value |  | ITP | healthy control | *Z* / *r* / *p*-value |
| IL-2 | 2.42 (1.69, 3.64) | 2.22(1.26, 4.95) | -1.149  / 0.119  /0. 881 |  | 2.45 (1.37, 5.59) | 1.59 (1.07, 4.46) | -0.917  / 0.165  /0.359 |
| IL-4 | 2.82 (1.61, 4.32) | 2.04 (1.15, 3.74) | -0.842  / 0.087  /0.400 |  | 3.24 (1.74, 4.50) | 1.34 (0.62, 4. 05) | -2.070  / 0.372  /0.038 |
| IL-6 | 13.84(4.15, 26.48) | 15.43 (6.68, 43.42) | -0.853  / 0.088  /0.394 |  | 9.24 (4.47, 23. 37) | 11.51 (3.93, 24.79) | -0.059  /0.011  /0.953 |
| IL-10 | 6.87(4.98, 11.17) | 5.44 (3.26, 9.07) | -1.503  / 0.155  /0. 133 |  | 6.16 (3.56, 11.39) | 3.16 (1.60, 6.08) | -2.203  /0.396  /0.028 |
| IL-35 | 312.81(135.85, 685.13) | 424.64(166.25, 1301.15) | -1.551  /-0.139  /0.121 |  | 133.95(17.20, 297.63) | 458.51(238.35, 765.59) | -2.122  /-0.190  /0.034 |
| IFN-γ | 2.89(1.76, 3.82) | 2.84(1.94, 9.76) | -0.352  /0.036  /0.725 |  | 2.72(1.89, 3. 62) | 1.93 (1.28, 5. 39) | -0.857  /0.154  /0.391 |
| TNF-α | 5.55(2.79, 18.01) | 3.99(1.88, 7.65) | -1.514  /0.156  /0.130 |  | 2.96(2.21, 6.40) | 2.35(0.93, 13. 09) | -1.050  /0.189  /0.294 |

* Mann-Whitney U test was applied. Effect size: *r*.
